# Primary Symptoms, Comorbidities, and Outcomes of 431 Hospitalized Patients with Confirmative RT-PCR Results for COVID-19

**DOI:** 10.4269/ajtmh.20-0512

**Published:** 2020-06-24

**Authors:** Amir Hossein Norooznezhad, Farid Najafi, Parisa Riahi, Mehdi Moradinazar, Ebrahim Shakiba, Shayan Mostafaei

**Affiliations:** 1 Medical Biology Research Center, Health Technology Institute, Kermanshah University of Medical Sciences, Kermanshah, Iran;; 2 Research Center for Environmental Determinants of Health (RCEDH), Health Institute, School of Health, Kermanshah University of Medical Sciences, Kermanshah, Iran;; 3 Department of Biostatistics, Faculty of Medical Sciences, Tarbiat Modares University, Tehran, Iran;; 4 Department of Biostatistics, School of Health, Kermanshah University of Medical Sciences, Kermanshah, Iran;; 5 Epidemiology and Biostatistics Unit, Rheumatology Research Center, Tehran University of Medical Sciences, Tehran, Iran

## Abstract

This study aimed to evaluate the primary symptoms, comorbidities, and outcomes of inpatients with confirmed reverse transcription–PCR (RT-PCR) for SARS-CoV-2 infection among 2077 suspected/diagnosed cases of COVID-19. Based on the results of Least Absolute Shrinkage and Selection Operator (LASSO) logistic regression, age, and suggestive chest X-ray (CXR) findings for SARS-CoV-2 infection, cardiovascular diseases, diabetes mellitus, chronic lung diseases, and intensive care units admission had significant associations with positive RT-PCR results for COVID-19 infection. Also, the highest area under the curve (AUC) was related to cough (AUC = 0.53, 95% CI: 0.51–0.56), dyspnea (AUC = 0.52, 95% CI: 0.50–0.54), and abnormal CXR (AUC = 0.52, 95% CI: 0.50–0.54), as significant predictors. This study showed that some symptoms including cough and dyspnea, as well as abnormal CXR, could be proper predictors of positive RT-PCR result for SARS-CoV-2 infection. It seems that patients with underlying disease(s), such as cardiovascular diseases, diabetes mellitus, and chronic lung diseases, had a higher probability to have positive RT-PCR for SARS-CoV-2 infection than those with no underlying disease(s).

## INTRODUCTION

In late 2019, a new coronavirus outbreak started in Wuhan (Hubei Province, China), which rapidly turned into a pandemic emergency according to the WHO, and, now (10:00 CEST, June 12, 2020), more than 7.4 million individuals have emerged as infected cases.^1^ The main feature of the disease is pneumonia,^2^ and most patients have primary symptoms such as fever, cough, dyspnea, sore throat, myalgia, gastrointestinal penetrations, and rhinorrhea.^3,4^ This study aimed to evaluate the possible predictive value of the most important symptoms and underlying diseases in patients diagnosed with COVID-19 using reverse transcription–PCR (RT-PCR) in three age-groups of children and adolescents, adults, and elderly.

## METHODS AND PATIENTS

This analytical cross-sectional study was performed in Farabi and Imam-Reza hospitals as two designated centers for COVID-19 patients hospitalization by the Ministry of Health in Kermanshah (a western province), Iran. The database used herein was obtained from the vice-chancellor of health in the Kermanshah University of Medical Sciences gathered from 2077 suspected/diagnosed cases of COVID-19 from February 20 to April 9, 2020. April 9 is the first day an official statement was made on the first confirmed COVID-19 case in Iran by the WHO.^5^ The inclusion criteria were defined as 1) individuals with symptom(s) in favor of COVID-19 and 2) inpatients with confirmative RT-PCR result for SARS-CoV-2 infection. Also, any lack of information in any patient led to their exclusion from the study.

Primary data on the hospitalized patients such as biodemographic information, presenting symptoms, underlying disease(s), and abnormal findings in chest X-ray (CXR) were investigated based on the LASSO logistic regression by “glmnet” R package. Moreover, ROC curve analysis was performed for the predicted diagnostic value of the already mentioned variables for prediction of a positive RT-PCR result for SARS-CoV-2 infection.

The data were supported and validated by the vice-chancellor of health in the Kermanshah University of Medical Science, and permission was granted to use the mentioned information only for research purposes, not as any official statics for the province. This study was approved by the Medical Ethics Committee of Kermanshah University of Medical Sciences. All the data were encrypted and de-identified when received from the mentioned source. Also, all authors declare their adherence to the declaration of Helsinki in 1964 and its further revisions.

## RESULTS

Among 2077 suspected/diagnosed cases and considering both inclusion and exclusion criteria, 431 individuals with a definite diagnosis for COVID-19 infection based on positive RT-PCR and 1,646 cases with negative RT-PCR results were enrolled in this study. Of those 431 inpatients, children and adolescents (aged ≤ 18 years), adults (aged between 18 and 65 years), and elderlies (aged ≥ 65) had the prevalence of 3.5% (15/431), 77.5% (334/431), and 19% (82/431), respectively, among which 29 (6.7%) were admitted to intensive care units (ICUs). The total fatality rate in RT-PCR positive group was 9% (39/431), in which children and adolescent, adult, and elderly groups have 0% (0/15), 4.8% (16/334), and 28.1% (23/82) proportions, respectively.

There was a significant association between variables, including age, abnormal CXR findings, cardiovascular diseases, diabetes mellitus, chronic lung diseases, and ICU admission, with confirmative RT-PCR results for SARS-CoV-2 infection. However, cough, dyspnea, elevated body temperature (both ≥ 37.7°C and ≥ 38.0°C), and rhinorrhea were among the most prevalent nonsignificant associated features with the confirmed result of RT-PCR for SARS-CoV-2 infection (*P*-value> 0.05). Also, significantly associated features in the evaluated patients by their age-groups are provided in Table 1.

**Table 1:** Association between selected patients’ characteristics with results of RT-PCR (431 cases with positive RT-PCR and 1,646 cases with negative RT-PCR results) by LASSO logistic regression

Associated variable	All patients (*n* = 431)	Children (*n* = 15)	Adult (*n* = 334)	Elderly (*n* = 82)
Age (years)	**94%, 0.03**	NA	**NA**	**NA**
Fever	80%, *P* = 0.28 (42%)	**71%, *P* = 0.33** **(40%)**	79%, *P* = 0.18 (35%)	**90%, *P* = 0.036** (**49%)**
Cough	89%, *P* = 0.07 (60%)	100%, *P* = 0.001 (69%)	**90%, *P* = 0.044** (**60%)**	70%, *P* = 0.09 (55%)
Dyspnea	85%, *P* = 0.13 (52%)	66%, *P* = 0.34 (18%)	**89%, *P* = 0.021** (**60%)**	**92%, *P* = 0.017** (**66%)**
Weakness	75%, *P* = 0.37 (9%)	68%, *P* = 0.78 (5%)	79%, *P* = 0.18 (6%)	**86%, *P* = 0.048** (**13%)**
Myalgia	–	–	63%, *P* = 0.37 (8%)	66%, *P* = 0.29 (12%)
Confusion	70%, *P* = 0.50 (2%)	–	75%, *P* = 0.46 (2%)	63%, *P* = 0.59 (3%)
Sore throat	67%, *P* = 0.63 (11%)	–	75%, *P* = 0.18 (12%)	–
Rhinorrhea	86%, *P* = 0.12 (2%)	–	80%, *P* = 0.25 (2%)	**93%, *P* = 0.035** (**4%)**
Diarrhea	82%, *P* = 0.15 (3%)	–	80%, *P* = 0.35 (2%)	83%, *P* = 0.25 (3%)
Nausea/vomiting	74%, *P* = 0.47 (5%)	–	76%, *P* = 0.33 (4%)	85%, *P* = 0.07 (6%)
Chest pain	80%, *P* = 0.28 (7%)	–	**88%, *P* = 0.031** (**6%)**	**86%, *P* = 0.048** (**9%)**
Body temperature ≥ 38°C	80%, *P* = 0.27 (26%)	81%, *P* = 0.19 (18%)	**86%, *P* = 0.038** (**21%)**	**95%, *P* = 0.001** (**30%)**
Body temperature ≥ 37.7°C	83%, *P* = 0.10 (27%)	81%, *P* = 0.19 (19%)	**89%, *P* = 0.021** (**22%)**	**95%, *P* = 0.001** (**31%)**
Abnormal chest X-ray	**95%, *P* = 0.03** (**20%)**	–	**99%, *P* = 0.001** (**20%)**	**92%, *P* = 0.019** (**18%)**
Cardiovascular diseases	**97%, *P* = 0.026** (**12%)**	–	**89%, *P* = 0.017** (**5%)**	**100%, *P* = 0.001** (**30%)**
Diabetes mellitus	**98%, *P* = 0.019** (**9%)**	–	**91%, *P* = 0.007** (**6%)**	**99%, *P* = 0.001** (**13%)**
Chronic kidney disease	75%, *P* = 0.37 (2%)	–	70%, *P* = 0.44 (1%)	78%, *P* = 0.31 (3%)
Chronic lung disease	**99%, *P* = 0.007** (**4%)**	–	**98%,** *P* = **0.001** (**3%)**	**98%, *P* = 0.001** (**6%)**
Malignancy	–	–	69%, *P* = 0.58 (2%)	66%, *P* = 0.67 (2%)
Intensive care unit admission	**100%, *P* = 0.005** (**11.6%)**	–	**100%, *P* = 0.001** (**6%)**	**97%, *P* = 0.001** (**14%)**

NA = not applicable; *P* = adjusted *P*-value using the methods by Benjamini and Hochberg (% relative frequency); RT-PCR = reverse transcription–PCR. Important values have been reported based on the *Z* score. Bold face values indicate statistical significance at the level of 0.05.

The highest area under the curve (AUC) was related to cough (AUC = 0.53, 95% CI 0.51–0.56), dyspnea (AUC = 0.52, 95% CI 0.50–0.54), and abnormal CXR (AUC = 0.52, 95% CI 0.50–0.54), as the most significant predictors of confirmed RT-PCR for SARS-CoV-2 infection. In adults and elderly patients, the highest AUC was related to abnormal CXR (Table 2).

**Table 2:** AUCs with 95% CI of clinical signs and symptoms for prediction of COVID-19 based on the results of RT-PCR (431 cases with positive RT-PCR and 1,646 cases with negative RT-PCR results) by univariate ROC curve analysis

Associated factor	All patients (*n* = 431)	Children (*n* = 15)	Adult (*n* = 334)	Elderly (*n* = 82)
Fever	0.52 (0.49–0.54)	0.51 (0.47–0.54)	0.52 (0.50–0.54)	0.51 (0.50–0.53)
Cough	**0.53** (**0.51–0.56)**	**0.52** (**0.51–0.57)**	**0.53** (**0.51–0.55)**	**0.505** (**0.50–0.54)**
Dyspnea	**0.52** (**0.50–0.54)**	**0.51** (**0.50–0.54)**	**0.52** (**0.50–0.54)**	**0.54** (**0.52–0.56)**
Weakness	0.50 (0.48–0.52)	0.50 (0.48–0.52)	0.50 (0.48–0.52)	**0.51 (0.50–0.53)**
Myalgia	0.50 (0.48–0.52)	0.50 (0.48–0.52)	0.50 (0.48–0.52)	0.50 (0.48–0.52)
Confusion	0.50 (0.47–0.53)	0.50 (0.45–0.54)	0.50 (0.47–0.53)	0.50 (0.47–0.53)
Sore throat	0.50 (0.48–0.53)	0.50 (0.48–0.53)	0.50 (0.48–0.53)	0.50 (0.48–0.53)
Rhinorrhea	0.50 (0.48–0.53)	0.50 (0.48–0.52)	0.50 (0.48–0.53)	**0.51 (0.50–0.54)**
Diarrhea	0.51 (0.48–0.53)	0.50 (0.48–0.52)	0.51 (0.48–0.53)	0.51 (0.48–0.53)
Nausea/vomiting	0.51 (0.48–0.52)	0.50 (0.48–0.52)	0.51 (0.48–0.52)	0.51 (0.48–0.52)
Chest pain	0.51 (0.48–0.53)	0.51 (0.49–0.53)	0.51 (0.48–0.53)	**0.52 (0.50–0.55)**
Body temperature ≥ 38°C	0.51 (0.46–0.54)	0.51 (0.45–0.54)	**0.52 (0.50–0.54)**	**0.52 (0.51–0.53)**
Body temperature ≥ 37.7°C	0.52 (0.48–0.55)	0.52 (0.47–0.55)	**0.51 (0.50–0.54)**	**0.52 (0.51–0.54)**
Abnormal chest X-ray	**0.52 (0.50–0.54)**	**0.51 (0.50–0.53)**	**0.55 (0.51–0.59)**	**0.54 (0.52–0.58)**

AUC = area under a ROC curve; 95% CI. Bold face values indicate statistical significance at the level of 0.05.

**Figure 1.**
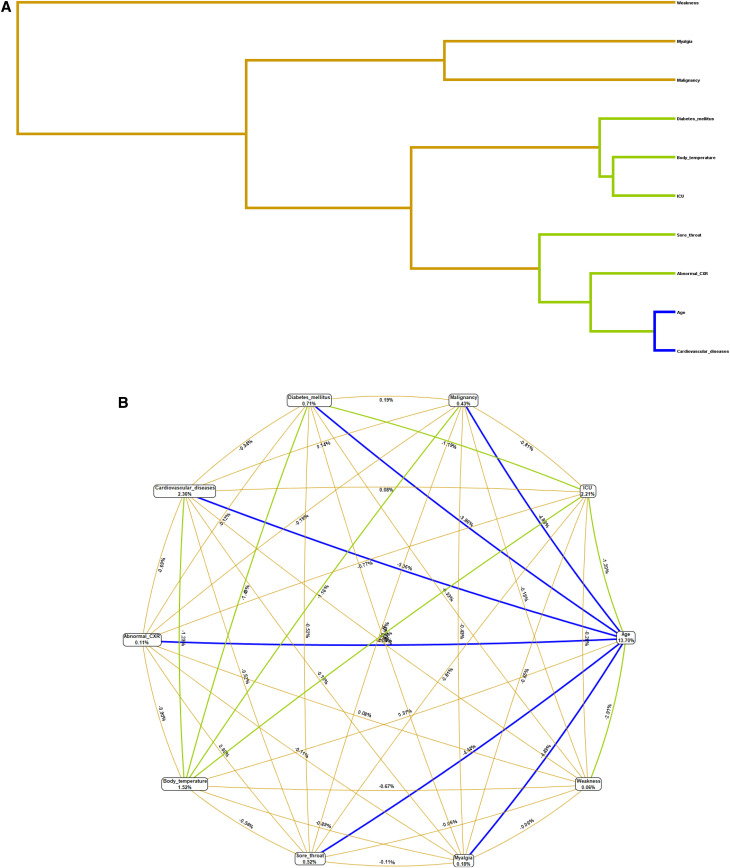
(**A**) Hierarchical dendrogram of multifactor dimensionality reduction (MDR) analysis. The shorter the line connecting the two attributes, the stronger the interaction effects on the death of positive RT-PCR patients. Blue color of the connecting line is indicative of the high degree of the redundancy (or most relative). In the attributes connecting with green line, these are of a less degree of redundancy in terms of interaction (or middle relative). Gold lines are representing the independent (or less relative) attributes, as their interaction coefficients are not significant. (**B**) Circle graph of MDR algorithm. Circle graph indicator of information gain as the main effects of each variable and the interaction effects between them to prediction of death among positive RT-PCR patients. This figure showed age, cardiovascular disease, and intensive care unit (ICU) admission have stronger main effect on the risk of death. Also, the interaction among age (older than 65 years), cardiovascular disease, diabetes mellitus, myalgia, malignancy, sore throat, and abnormal chest X-ray (CXR) had stronger effects on death among positive RT-PCR patients.

In Figure 1A, the hierarchical dendrogram, age, and cardiovascular diseases have the strongest interactions compared with the other variables for the prediction of death among positive RT-PCR patients. The color of the connecting line, blue, is indicative of the high degree of redundancy (or most relative). Considering the attributes connecting with the green line, these are of a less degree of redundancy in terms of interaction (or middle relative). Gold lines are representing the independent (or less relative) attributes, as their interaction coefficient is not significant. Also, the circle graph (Figure 1B) was used as the indicator of interaction network for the death predictors based on the information gain (IG) of each attribute, also considered as the main effect value. As it is shown (Figure 1B), age (IG = 13.7%), cardiovascular disease (IG = 2.4%), and ICU admission (IG = 2.2%) have the most important main effects, and they have interaction effects with abnormal CXR, elevated body temperature, myalgia, sore throat, diabetes mellitus, and malignancy for the prediction of death among positive RT-PCR patients.

## DISCUSSION

The results showed that the fatality rate was higher in elderly than other age-groups. Also, underlying diseases including cardiovascular diseases, diabetic mellitus, and chronic lung diseases were significantly associated with a positive RT-PCR result for SARS-CoV-2 infection. Changes in the importance of variables in each age category showed the importance of each one as clinically important parameters in suspected COVID-19 cases. Also, Table 2 shows that cough, dyspnea, and abnormal CXR seem to be proper tools for the prediction of confirmed RT-PCR in cases suspected of COVID-19. We have chosen only RT-PCR–positive cases because at the beginning of the outbreak, details on radiologic findings were not as well known as those at the end of the study, which might lead to false-positive or negative result in our evaluated patients. Thus, including only RT-PCR–positive patients might be one of the reasons why the outcome ratios such as fatality rate or ICU admission could not be relied on as full population-based statics.

According to Tables 1 and 2, some variables seem to be associated with a positive RT-PCR result in symptomatic cases. On the other hand, it has been shown that RT-PCR is a confirmative diagnostic method for COVID-19, with a sensitivity of 71%. Considering the study by Fang et al.,^6^ who evaluated symptomatic patients, it has been shown that 36/51 of their cases had the primary confirmed RT-PCR results for SARS-CoV-2 infection on the first test. They performed second and third tests on days 1–2 and 2–5, which were confirmative for 12/51 (cumulative 48/51) and 2/51 (cumulative 50/51) of their patients, respectively. For the remaining patients, RT-PCR for SARS-CoV-2 confirmed COVID-19 infection on day 7 (cumulative 51/51),^6^ whereas the cumulative ratio for abnormal chest computed tomography (CT) scan at the first day was 50/51 as they have reported.^6^ Considering their result as well as having a glance at Tables 1 and 2, when RT-PCR is negative for patients with already mentioned variables (bolded ones), two pathways could be lead: 1) repeating RT-PCR on the following days and 2) requesting a chest CT scan with a sensitivity of 97% for the diagnosis of COVID-19.^7^ However, because some patients might have at least an underlying disease, affecting lungs (such as cardiovascular disease and chronic lung disease), we suggest not to miss following up these patients by further RT-PCR tests.^8^

Regarding the symptoms, a systematic review and meta-analysis on patients diagnosed with COVID-19 has shown that fever (88.7%), cough (57.6%), and dyspnea (45.6%) were the most prevalent symptoms in the patients (regardless of age); however, no subgroup for the method of diagnosis has been investigated. From all the symptoms evaluated in the mentioned review, authors have just shown differences of fever and cough among adults and children (fever: 92.8% versus 43.9%; cough: 63.4% versus 22% respectively). However, no other subgroup analysis has been performed neither for children nor for the elderlies.^3^ Another study evaluating the findings of two groups of young (*n* = 38) and elderly (*n* = 18) cases with confirmed COVID-19 infection showed that there was no difference between the prevalence of fever, cough, dyspnea, fatigue, rhinorrhea, or vomiting between the two groups.^4^ Thus, it seems that this study has added some new findings regarding a large number of confirmed COVID-19 patients in three age-groups by their symptoms.

## CONCLUSION

Altogether, we investigated the importance of primary findings in the patients who had a definite laboratory diagnosis for COVID-19 (RT-PCR) in three age categories including children and adolescents, adults, and the elderlies. The presence of some presenting symptoms and/or underlying diseases seems to be associated with a confirmed RT-PCR result for SARS-CoV-2 infection depending on the age category. We are certainly following the performed progress in the diagnosis/outcomes of patients with COVID-19 after the end of period for the current study to assess the quality of experiences earned during the time.
